# Synthesis and Characterization of bis[(2-ethyl-5-methyl-imidazo-4-yl)methyl]Sulfide and Its Coordination Behavior toward Cu(II) as a Possible Approach of a Copper Site Type I

**DOI:** 10.1155/2009/905320

**Published:** 2009-07-02

**Authors:** Juan D. Barrón-Garcés, Guillermo Mendoza-Díaz, Florina Vilchez-Aguado, Sylvain Bernès

**Affiliations:** ^1^Laboratorio de Química Bioinorgánica, Departamento de Química, División de Ciencias Naturales y Exactas, Universidad de Guanajuato, Noria Alta S/N, 36050, Guanajuato, GTO, Mexico; ^2^Departamento de Química, División de Ciencias Naturales y Exactas, Universidad de Guanajuato, Unidad Cerro de la Venada S/N, 36040 Guanajuato, GTO, Mexico; ^3^División de Estudios de Posgrado (DEP), Facultad de Ciencias Químicas, Universidad Autónoma de Nuevo León, Guerrero y Progreso S/N, Colonia Treviño, 64570, Monterrey, NL, Mexico

## Abstract

The synthesis and characterization of a novel ligand, bis[(2-ethyl-5-methyl-imidazo-4-yl)methyl]sulfide (*bemims*), as well as a *bemims*-containing copper(II) coordination complex are described. In this complex, [Cu(*bemims*)*X*_2_] with *X* = NO_3_ ** ^−^, *bemims* acts as a tridentate ligand and two monodentate nitrate ions complete the coordination sphere. Both imidazole N atoms and the thioether S atom of *bemims* participate in coordination. The Cu(II) ion is five-coordinated with a slightly distorted square-pyramidal geometry (*τ* = .09). Electrochemical studies and spectroscopic data for this complex are compared with some blue copper proteins in order to assess its ability to mimic the copper center of type I copper proteins.

## 1. Introduction

**Figure 1:**
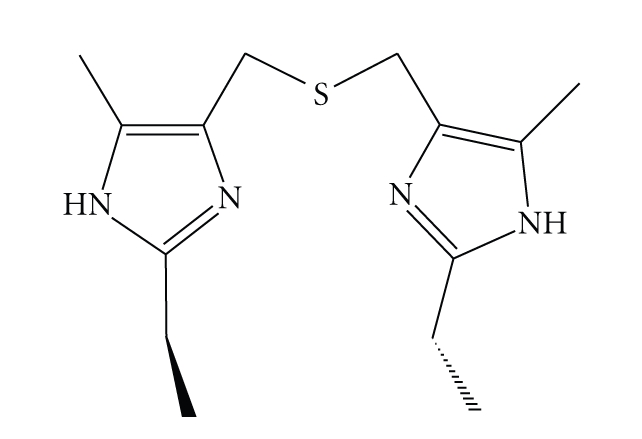
Schematic drawing of the ligand *bemims*.

Copper is an essential element for life, as it actively participates in many biological events [1]. This transition metal is the second important element involved in electron transfer processes [2–4], and copper ions present in proteins play a crucial role as electron carriers in many vital processes. The large family of copper proteins includes one group of particular interest, the so-called type I or “blue” copper proteins (T1Cu). A number of publications, either experimental or theoretical, have been devoted to T1Cu proteins, due to their special features, such as their ability to transport electrons over large distances and hence to catalyze selected chemical reactions. Copper proteins possess well-designed active centers that finely tune metal ion redox properties [5, 6]. In the case of T1Cu proteins, the active site contains two imidazole N atoms from histidine residues, one thioether S atom from methionine and one thiolate S atom from cysteine. Blue copper proteins active sites may display tetrahedral geometry with two N and two S donor atoms, although five-coordinated Cu(II) centers with a trigonal bipyramidal geometry were also found, for example, in azurins [7]. More than a couple of decades ago, many groups synthesized ligands containing imidazole derivatives and thioether functionalities, with the hope to obtain copper complexes suitable as models for T1Cu active sites. Despite numerous efforts, such a molecular model is not yet available. However, research keeps growing on this field. As a contribution, the present work describes the synthesis and characterization of a novel ligand, namely, bis[(2-ethyl-5-methyl-imidazo-4-yl)methyl]sulfide (*bemims*), (see Figure 1) and its complexation to Cu(II), as a possible approach for a model of an active site of a T1Cu protein.

## 2. Experimental Section

### 2.1. Materials

Initial material 2-ethyl-4-methyl-5-hydroxymethylimidazole hydrochloride was synthesized as described in [8], and all other chemicals, including sodium sulfide nonahydrate and cupric nitrate trihydrate, were purchased and used without previous purification.

### 2.2. Synthesis of bis[(2-ethyl-5-methyl-imidazo-4-yl)methyl]sulfide (*bemims*, Figure 1).

An amount of 2-ethyl-4-methyl-5-hydroxymethylimidazole hydrochloride (10 g, 0.0566 mol) was dissolved in 10 mL of water at room temperature. An aqueous solution (10 mL) of Na_2_S · 9H_2_O (22.16 g, 0.0923 mol) was added slowly to the imidazole derivative solution. The reaction mixture was stirred for 48 hours, giving a yellow solution with a white precipitate. The solvent was removed by filtration and the solid portion was washed with cool water. The dried solid product was dissolved in methanol and purified by column chromatography with silica-gel as support, using a mixture (20:80) methanol/chloroform as eluent. To the resulting solution, ethyl acetate was added, and finally, solvents were removed in a vacuum line, affording a microcrystalline white solid. This solid product was recrystallized from a methanol/water mixture. 

Yield: 9.45 g (0.0339 mol, 90%). M.p. 200–20°C. ^1^H-NMR (CDCl_3_, 200 MHz,*δ*/ppm): 1.3 (t, 6H, CH_3_), 2.1 (*s*, 6H, CH_3_), 2.7 (c, 4H, CH_2_), 3.5 (s, 4H, CH_2_), 9.3 (broad, 2H, NH). ^13^C-NMR (CDCl_3_, 50 MHz *δ*/ppm): 10.79 (CH_3_-CH_2_), 12.69 (CH_3_-Imi), 21.94 (CH_2_-CH_3_), 24.70 (CH_2_-S), 127.11 (C5-Imi.), 127.76 (C4-Imi.), 147.42 (C2-Imi). 

NMR spectra were recorded on a Varian Gemini 2000 (200 MHz) spectrometer.

### 2.3. Synthesis of the Copper Complex

The coordination compound was prepared by dissolving CuNO_3_ · 3H_2_O (1.74 g, 0.0072 mol) in 10 mL of methanol, which was added to a solution of the ligand *bemims* (2 g, 0.0072 mol) in 10 mL of methanol. The reaction mixture was stirred for 20 minutes at room temperature, resulting in a green solution with a microcrystalline precipitate of the same color. The complex was filtered off and recrystallized from a methanol solution at 10°C over several days. 

Yield: 85% (2.85 g, 0.0061 mol). Molar Conductivity (**Λ**_**M**_), was measured in a 10^−3^ M methanol solution **Λ_M_** = 76.91 ohm^−1^ cm^2^ mol^−1^, Magnetic Susceptibility was measured in a Sherwood Scientific CB18DH Gouy balance at room temperature.

### 2.4. Spectroscopic Measurements

A methanol solution of the copper complex (8.58 10^−3^ M) was used. The electronic spectrum in visible region was recorded on an Agilent HP 8553 spectrophotometer, using quartz cells with 1 cm optic path length. IR spectra (KBr pellets) were taken using a Perkin Elmer 1600 FTIR spectrometer in the 400 to 4000 cm^−1^ frequencies interval. All experiments were carried out at room temperature.

### 2.5. Electrochemical Measurements

Cyclic voltametry was performed in a three-electrode cell consisting of a glassy carbon electrode (BAS) of 3 mm diameter, a platinum wire as counter electrode, and an Ag/AgCl/KCl_sat_ reference electrode. The working electrode, before use, was polished over a microcloth (Buehler 40-7218) with alumina particles (Buehler), of three sizes, 1, 0.3, and 0.05 *μ*m, and washed with desionized water. The cyclic voltametry experiment was carried out at room temperature and under N_2_ atmosphere, using a tetrabutylammonium tetrafluoroborate solution (4.8 × 10^−2^ M) as support electrolyte. An Elda300 potentiostat, from Radiometer Analytical S.A. Tacussel, was used.

### 2.6. Diffraction Data and Structure Refinement

X-ray diffraction intensities for complex [Cu(*bemims*)(NO_3_)_2_] were collected on a Siemens P4 diffractometer, equipped with a normal focus Mo-K*α* X-ray tube (*λ* = 0.71073 Å) operated at 1250 W. Data were processed with XSCANS [9] and corrected for absorption effects on the basis of Ψ-scans data. The structure was solved and refined using the SHELX programs [10]. All non-H atoms were refined anisotropically and H atoms were placed in idealized positions and refined as riding to their carrier atoms. Atom S1 is disordered by symmetry over two sites, and its occupancy in the asymmetric unit was fixed to 1/2. A summary of essential crystallographic parameters may be found in Table 1 while complete data are available from the deposited CIF.

## 3. Results and Discussion

### 3.1. Spectroscopy

The copper complex [Cu(*bemims*)(NO_3_)_2_] shows an absorption broad band at *λ*_max_ = 655 nm (*ε* = 121.93 M^−1^cm^−1^), due to *d-d * transitions. Five-coordinated Cu(II) complexes showing absorption in the 588–769 nm region approximate a square-pyramidal geometry, while complexes with a trigonal-bipyramidal geometry show absorption bands in the 685–952 nm region, with highest absorption intensities in the range 666-877 nm [11]. The electronic spectrum of [Cu(*bemims*)(NO_3_)_2_] is thus in agreement with a square-pyramidal [Cu(II)SN_2_O_2_] coordination sphere, which is confirmed by structural studies (see *infra*). Measured conductivity indicates that in methanol the nitrates are coordinated and therefore the complex is a no electrolyte. The characteristic infrared vibrations for the free ligand and the complex were easily assigned. Ligand *bemims* shows four characteristic vibrations at 1609, 1533, 1449, and 1428 cm^−1^, due to C=C and C=N(imidazole) stretching vibrations, respectively, and one vibration at 1044 cm^−1^, corresponding to the C–S stretching vibration. In the Cu(II) complex, vibrations are shifted to 1637, 1544, 1474, and 1416 cm^−1^ for imidazole stretching vibrations, and to 1032 cm^−1^ for the C–S bonds [12]. Additional vibration at 1300 cm^−1^ appears, assigned to N–O stretching of the nitrate ligands [13]. In solid state magnetic susceptibility was measured at room temperature, giving a magnetic moment of *μ*_eff_ = 2.216 BM, which is in the normal range for Cu(II) complexes with no magnetic interaction in the lattice.

### 3.2. Electrochemical Results

**Figure 2:**
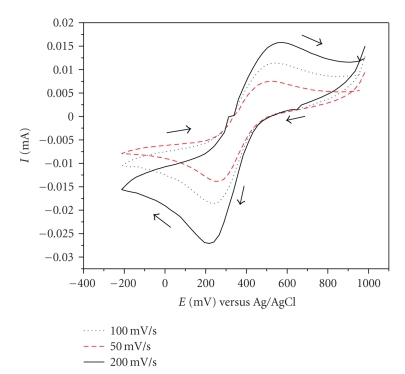
Cyclic voltammogram recorded at a glassy carbon electrode in CH_3_CN containing [Cu(*bemims*)(NO_3_)_2_] (2.9 10^−2^ M) and tetrabutylammonium tetrafluoroborate solution (4.8 10^−2^ M). Scan rate 50–200 V*s* ^−1^.

In the copper complexes, the redox couple Cu(II)/Cu(I) is important and is known to be strongly influenced by ligand factors, such as the nature of donor atoms and their structural arrangement around the copper ion [14, 15]. Figure 2 shows the cyclic voltammogram of the complex [Cu(*bemims*)(NO_3_)_2_] dissolved in acetonitrile. Scans were carried out in the cathodic direction at different rates, 50, 100, and 200 mV*s* ^−1^, which did not alter significantly the peak positions and *E*_1/2_. The redox process Cu(II) ↔ Cu(I) is a quasireversible process: by increasing the scan rate, the ratio * I*_pa_/*I*_pc_ approaches 1. Due to the low solubility of the complex in aqueous solution, the redox potentials were measured in acetonitrile, and it is thus not be possible to compare directly these values (Table 2) with those of T1Cu proteins, measured in aqueous solution. However, the high redox potential observed for [Cu(*bemims*)(NO_3_)_2_] is in line with high potentials (more than 250 mV) characterizing almost all T1Cu proteins.

### 3.3. Crystal Structure

**Figure 3:**
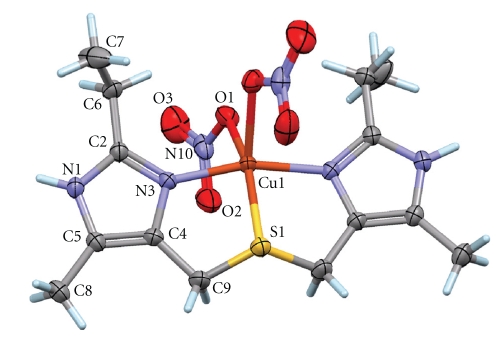
ORTEP projection [16] of the complex [Cu(*bemims*)(NO_3_)_2_] with displacement ellipsoids for non-H atoms at the 30% probability level. The labeling scheme is given for the asymmetric unit, while unlabelled atoms are generated by symmetry 1-*x*, *y*, 1/2-*z*. For clarity, a single disordered site for S1 is shown.

The ORTEP projection with the atomic labeling scheme for [Cu(*bemims*)(NO_3_)_2_] is displayed in Figure 3. The *bemims* ligand acts as a tridentate ligand and two monodentate nitrate ions complete the coordination, affording a neutral complex. The complex lies on a two-fold axis, resulting in an asymmetric unit containing one-half complex (*Z*′= 1/2). However, the S atom of the *bemims* ligand is placed in general position, and is thus disordered over two equally occupied sites through the two-fold axis. Such a disorder is consistent with a rapid inversion of the tetrahedral S atom, with the lone pair disordered above and below the mean plane of *bemims*. Interestingly, a closely related complex was previously X-ray-characterized [17], which does not present such an inversion: by formally substituting *bemims* by bis[(5-methyl-imidazo-4-yl)methyl]sulfide, *bmims*, a similar complex may be synthesized, [Cu(*bmims*)(NO_3_)_2_], which crystallizes with the whole molecule placed in general position, and with the tetrahedral thioether S atom in a freezing conformation. This difference between closely related complexes has an interesting consequence on the description of the coordination geometry around Cu(II). In the case of [Cu(*bmims*)(NO_3_)_2_], the Cu(II) ion has a distorted square-pyramidal geometry, with the apical site occupied by a nitrate O atom [17]. The distortion parameter for this nondisordered complex is *τ* = 0.24 (where *τ* is defined as *τ* = (*θ* − *φ*)/60; with *θ* and *φ* being the two largest coordination angles; *τ* = 0 for a perfect square-pyramidal geometry; *τ* = 1 for a perfect trigonal-bipyramidal geometry [18]). For the here reported [Cu(*bemims*)(NO_3_)_2_] complex, *τ* = 0.09 taking into account one disordered site for the thioether S atom. The coordination environment for Copper(II) should then be considered that is closer to square-pyramidal geometry. With this description, one nitrate O atom occupies the apical position, and the symmetry-related nitrate is placed in the base of the pyramid. However, an alternative description is to consider that, due to tetrahedral inversion, the actual mean position for the thioether S atom is at the midpoint between the two crystallographically equivalent positions. The computed distortion parameter is then *τ* = 0.42, reflecting a strong tendency to the trigonal-bipyramidal geometry, with axial positions defined by imidazole N atoms. In other words, the actual distortion for the coordination geometry in [Cu(*bemims*)(NO_3_)_2_] cannot be unambiguously determined, and would be difficult to predict in solution, where solvent and dilution effects probably determine the rate of inversion for the thioether S atom. This flexible behavior may explain why broad absorptions are observed in the electronic spectra.

Although [Cu(*bemims*)(NO_3_)_2_] strongly deviates from a perfect geometry to be a good model for a T1Cu protein active site, some structural features compare well with those of a couple of T1Cu proteins, azurin from *Alcaligenes denitrificans * and poplar plastocyanin. Coordination bond lengths for these proteins [7] and [Cu(*bemims*)(NO_3_)_2_] are collected in Table 3 for comparison purpose.

## 4. Conclusions

The synthesis of the novel ligand containing imidazole derivatives and thioether has been successful. *Bemims* compound acts as tridentate ligand toward Cu(II). The copper complex obtained is five-coordinated with a flexible square-pyramidal geometry, solid state as in methanol solution, according to spectroscopic measurements and structure determination. Redox studies point out to a quasireversible Cu(II)/Cu(I) system. The complex [Cu(*bemims*)(NO_3_)_2_] acts as an electroactive species showing a relatively high redox potential in acetonitrile. On the other hand, the bond lengths found in the complex show some similarity with those characteristic of some active sites of T1Cu proteins, for example, the active site of poplar plastocyanin. Complex [Cu(*bemims*)(NO_3_)_2_] may thus be considered as a potential candidate for further probes dealing with molecular model design of T1Cu active sites.

## Figures and Tables

**Table 1:** Crystallographic Data for [Cu(*bemims*)(NO_3_)_2_].

Compound	[Cu(*bemims*)(NO_3_)_2_]
Empirical formula	C_14_H_22_CuN_6_O_6_S
Formula weight	465.98
Colour, habit	Green prism
Crystal size [mm]	0.40 × 0.40 × 0.20
Space group	*C*2/*c*
*a * [Å]	8.4305(16)
*b * [Å]	15.354(3)
*c * [Å]	15.258(3)
**β** [º]	103.322(8)
*V * [Å^3^]	1921.9(6)
*Z*	4
*ρ*_calcd_ [g.cm^−3^]	1.610
*μ*[mm^−1^]	1.290
2*θ* Range [°]	5.5–52.5
Reflections collected	3976
Independent reflections (*R*_int_)	1937 (0.024)
Transmission factors [min., max.]	0.613, 0.772
Final *R * indices [*I* > 2*σ*(*I*)] *R*_1_, *wR *_2_	0.034, 0.084
Final *R * indices (all data)* R*_1_, *wR *_2_	0.040, 0.088
Goodness-of-fit on *F * ^2^	1.057
Data/restraints/parameters	1937/0/134
Largest difference peak/hole [e. Å^−3^]	0.445, −0.406

*R*_int_, *R *_1_ and *wR *_2_ are defined as follows: $R_{\text{int}}=\sum \left|F_{o}^{2}-\langle F_{o}^{2}\rangle \right|/\sum F_{o}^{2},\;R_{1}=\sum \left|\left|F_{o}\right|-\left|F_{c}\right|\right|/\sum \left|F_{o}\right|,wR_{2}=\sqrt{\sum w{\left(F_{o}^{2}-F_{c}^{2}\right)}^{2}/\sum w{\left(F_{o}^{2}\right)}^{2}}.$

**Table 2:** Redox properties of complex in [Cu(*bemims*)(NO_3_)_2_] CH_3_CN at 25°C. Scan rate 50–200 mVs^−1^, *E* vs Ag/AgCl.

Scan Rate (Vs^−1^)	*E*_pa_ (mV)	*E*_pc_ (mV)	*E*_1/2_ (mV)	**∆** *E*_*p*_ (mV)	*I*_pa_ (mA)	*I*_pc_ (mA)	*I*_pa_/*I*_pc_
							
50	518	262.5	390.25	255.5	0.0075	−0.0116	0.64
100	541	240	390.5	301	0.0115	−0.0145	0.79
200	565	216	390.5	349	0.016	−0.020	0.80

**Table 3:** Comparison of coordination bond lengths for two copper site type I and complex [Cu(*bemims*)(NO_3_)_2_].

	Azurin	Plastocyanin		[Cu(*bemims*)(NO_3_)_2_]
Bond	Distance (Å)		Bond	Distance (Å)

Cu–O(Gly)	3.13		Cu–O1(NO3)	2.1269(19)
Cu–N(His)	2.08	2.04	Cu–N3(Imi)	1.9541(19)
Cu–S(Cys)	2.15	2.13	Cu–O1(NO3)	2.1269(19)
Cu–N(His)	2.00	2.10	Cu–N3(Imi)	1.9541(19)
Cu–S(Met)	3.11	2.90	Cu–S1(Thiother)	2.4122(13)
